# Association of Cost Savings and Surgical Quality With Single-Vendor Procurement for Spinal Implants

**DOI:** 10.1001/jamanetworkopen.2019.15567

**Published:** 2019-11-15

**Authors:** Collin W. Blackburn, Nicolas R. Thompson, Joseph E. Tanenbaum, Allen J. Passerallo, Thomas E. Mroz, Michael P. Steinmetz

**Affiliations:** 1 Center for Spine Health, Cleveland Clinic, Cleveland, Ohio; 2 Case Western Reserve University School of Medicine, Cleveland, Ohio; 3 Department of Quantitative Health Sciences, Cleveland Clinic, Cleveland, Ohio; 4 Neurological Institute Center for Outcomes Research and Evaluation, Cleveland Clinic, Cleveland, Ohio; 5 Department of Population and Quantitative Health Sciences, Case Western Reserve University, Cleveland, Ohio; 6 Department of Supply Chain Management, Cleveland Clinic, Cleveland, Ohio; 7 Department of Orthopaedic Surgery, Cleveland Clinic, Cleveland, Ohio; 8 Department of Neurosurgery, Cleveland Clinic, Cleveland, Ohio; 9 Cleveland Clinic Lerner College of Medicine, Cleveland, Ohio

## Abstract

**Importance:**

Significant cost savings can be achieved from consolidating purchases of spinal implants with a single vendor. However, it is currently unknown whether sole-source contracting or vendor rationalization more broadly affects patient care.

**Objectives:**

To describe the single-vendor procurement of spinal implants, characterize the economic benefits of sole-source contracting, and gauge whether vendor rationalization is associated with a diminished quality of care.

**Design, Setting, and Participants:**

This retrospective cohort study assessed adult patients receiving single-level lumbar interbody fusions at a single institution from January 1, 2009, to July 31, 2017. Exclusion criteria included multilevel fusions and prior spinal fusions.

**Exposures:**

Patients were analyzed based on the number of vendors available to surgeons at the time of the patient’s surgery. January 1, 2009, to December 31, 2010, was defined as the multivendor period (10 vendors); January 1, 2011, to December 31, 2014, was defined as the dual-vendor period; and January 1, 2015, to July 31, 2017, was defined as the single-vendor period.

**Main Outcomes and Measures:**

Rates of 12-month revision surgery, complications, 30-day readmissions, and postoperative patient-reported outcomes, as measured by 5-dimension European Quality of Life (EQ-5D) and Patient-Reported Outcomes Measurement Information System-Global Health (PROMIS-GH) utilities. Propensity score weighting was performed to control for confounding. The Holm method was used to correct for multiple testing. Annual cost savings associated with the dual-vendor and single-vendor periods were also reported.

**Results:**

A total of 1373 patients (mean [SD] age, 59.2 [12.6] years; 763 [55.6%] female; 1161 [84.6%] white) were analyzed. Rates of revisions after adjusting for confounding were 3.2% (95% CI, 1.5%-6.7%) for the multivendor period, 4.5% (95% CI, 3.1%-6.5%) for the dual-vendor period, and 3.0% (95% CI, 1.7%-5.0%) for the single-vendor period. Complication rates were 5.3% (95% CI, 2.7%-10.1%) for the multivendor period, 7.2% (95% CI, 5.4%-9.6%) for the dual-vendor period, and 6.4% (95% CI, 4.6%-8.8%) for the single-vendor period. Readmission rates were 14.2% (95% CI, 9.7%-20.2%) for the multivendor period, 12.6% (95% CI, 10.1%-15.5%) for the dual-vendor period, and 9.7% (95% CI, 7.4%-12.7%) for the single-vendor period. Revisions, complications, and patient-reported outcomes were statistically equivalent across all periods. Readmissions were not statistically equivalent but not statistically different. The savings attributable to vendor rationalization were 24% for the dual-vendor and 21% for the single-vendor periods.

**Conclusions and Relevance:**

The single-vendor procurement of spinal implants was associated with significant cost savings without evidence of an associated decline in the quality of care. Large hospital systems may consider sole-source purchasing as a viable cost reduction strategy.

## Key Points

**Question:**

Are single-vendor models of purchasing spinal implants associated with lower hospital costs or diminished surgical quality compared with multivendor models?

**Findings:**

In this retrospective cohort study of 1373 adult patients, the marginal benefit of single-vendor procurement compared with dual-vendor procurement was a 21% reduction in the cost of spinal implants. Surgical quality, as measured by rates of revision operations, complications, readmissions, and patient-reported outcomes, did not show evidence of deterioration as the vendor base decreased from 10 to 1.

**Meaning:**

Consolidating purchases of spinal implants with a single vendor may generate incremental cost savings without compromising patient care.

## Teaser

This cohort study assesses whether single-vendor procurement of spinal implants is associated with reduce hospital expenditures without compromising surgical quality.

## Introduction

The cost of health care delivery in the United States is $3.5 trillion or approximately 18% of gross domestic product.^1^ Within this high-cost environment, spinal fusion surgery has been identified as 1 of the largest and fastest-growing medical expenditures.^2,3^ A single 30-day episode of spinal fusion surgery may incur more than $25 000 in total hospital costs.^4,5,6^ Surgical implants account for the most significant portion of these expenditures, composing almost half of the costs linked directly to patient care.^7,8^ Implant prices have been reported to be in the range of $400 to $1843 for a single pedicle screw, $540 to $2388 for an anterior cervical plate, and $938 to $7200 for a transforaminal lumbar interbody fusion cage, underscoring the wide variability in prices that contributes to the high mean cost of spinal implants.^7^ The benefit of such price variability is that average buyers can achieve significant savings by renegotiating more competitive prices with their vendors. For example, Robinson et al^9^ reported that a group of 10 West Coast hospitals could have reduced their cost of spinal fusion surgery by more than 40% by adopting local best practices in device purchasing.

Targeting this opportunity for savings, our institution implemented a 2-pronged strategy in 2011 for lowering the cost of spinal implants. First, a multivendor model (10 vendors) was abandoned in favor of a dual-vendor model for purchasing commodity (ie, nonspecialized) implants, such as pedicle screws, rods, and anterior cervical plates. By leveraging the significant scale and scope of our operations, our institution was able to negotiate volume-based discounts from 2 preferred suppliers. Second, a capitated pricing strategy was implemented to lower the cost of specialty devices and products used in minimally invasive surgery (MIS), which requires specialized instruments and cameras to be passed through small incisions.^10,11^ Existing vendors of specialty and/or MIS devices were persuaded to accept predetermined price ceilings established for different classes of devices. The purpose of this dual approach was to protect physician autonomy in choosing nonstandard or specialty devices while generating cost savings through discounts on commodity implants. In 2015, because of ongoing budgetary constraints, our institution furthered its cost reduction efforts by advancing to a sole manufacturer of commodity devices and renegotiating specialty and/or MIS devices under a revised pricing matrix.

Cost savings programs that target expenditures on medical devices have been described previously in the literature, especially in the field of orthopedics.^12,13,14,15,16,17,18,19,20,21,22,23,24,25^ To our knowledge, however, no studies have reported on cost savings in spinal surgery associated with a single-vendor procurement model. The objectives of this study were to describe our experience with the single-vendor procurement of spinal implants, characterize the economic benefit of sole-source contracting, and examine whether vendor rationalization, also known as supply base reduction, is associated with a diminishing quality of care.

## Methods

This study received institutional review board approval and a waiver of informed consent from the Cleveland Clinic. A waiver of consent was granted because this study was retrospective in nature and did not in any way affect patient care. This study followed the Strengthening the Reporting of Observational Studies in Epidemiology (STROBE) reporting guidelines.

### Preferred-Vendor Selection and Capitated Price Negotiations

The process of evaluating and awarding vendor contracts for commodity devices was performed by a committee exclusively composed of physicians (T.E.M. and M.P.S.). All surgeons performing spine operations were invited to participate in the committee. One physician champion was selected among the senior leaders of our spine center and partnered with supply chain representatives (A.J.P.) to coordinate the process. Participating surgeons were required to disclose financial conflicts of interest, including those of their immediate relatives, at the outset of the process. Surgeons receiving more than $5000 annually from a supplier of spinal implants were allowed to participate in meetings but not allowed to vote on the final outcome. The scope of devices to be defined as commodities, predominately surgical plates, screws, rods, and interbody cages, was determined by the physician committee. The committee reviewed historical purchases by vendor and line item and agreed on the volume and mix of implants needed for the future. The committee also established minimum customer service requirements, including the availability of operating room support from sales representatives. Device manufacturers were invited to submit competitive bids that detailed product availability, price, customer service levels, and expectations regarding the size of the total financial opportunity. After receipt of the bids, supply chain representatives further negotiated prices. Pricing was negotiated as fixed with no opportunities for price increases during the contract period. Competitive bids were selected based on product-match rates and presented to the physician committee in a deidentified format to ensure objectivity. Vendors were selected and awarded contracts by a vote of the surgeon committee. The physicians who cast votes were a mix of neurosurgeons and orthopedic spine surgeons, and most were fellowship trained. The 2011 and 2015 iterations of vendor negotiations were run using nearly identical operations.

Concurrent with this process, the prices of specialty and/or MIS devices were renegotiated using a capitated pricing strategy in which price ceilings were assigned to different implant line items regardless of technology or manufacturer. Price ceilings were established for each line item based on estimates of prevailing market rates, which were derived from price data obtained from an external benchmarking service. Manufacturers were given the opportunity to accept or decline the revised prices. Declining excluded the manufacturer from doing future business with our organization.

After selection, awarded vendors conducted a 45- to 60-day training and education program for physicians and nurses to ensure familiarity with new products before patient use. Product fairs, cadaver laboratories, and 1-on-1 reviews were offered. An oversight committee of nonconflicted surgeons was established to review requests for devices manufactured by nonapproved vendors. Exceptions are granted for proprietary products that offer specific clinical advantages over approved devices. To date, 6 exceptions have been granted. Adherence to purchasing contracts is monitored at the physician level. Nonadherent physicians are approached by supply chain representatives to assess potential concerns about the portfolio of approved products. Persistent nonadherence is elevated to the physician champion, and each case is handled individually.

### Clinical Outcomes

To investigate our institution’s quality of care, we retrospectively identified all patients who received a single-level lumbar interbody fusion (*Current Procedural Terminology* codes 22630 and 22633) at our institution from January 1, 2009, to July 31, 2017. We excluded patients younger than 18 years, those with a prior spinal fusion (instrumented or in situ), and those receiving a multilevel or anterior interbody fusion. Patients receiving transforaminal or posterior lumbar interbody fusion with posterolateral fusion were included unless the posterolateral fusion exceeded 1 level. All operations were performed on an inpatient basis. This population was chosen as a proxy for the overall quality of care at our spine center, which is composed of 14 hospital-employed surgeons performing more than 3500 cases annually, because of the relative homogeneity and high volume of cases.

Patients were stratified into 3 cohorts (multivendor, dual vendor, and single vendor) based on the date of their index surgery (January 1, 2009, to December 31, 2010; January 1, 2011, to December 31, 2014; or January 1, 2015, to July 31, 2017, respectively), and propensity matching was performed on the following variables obtained from the electronic health record: date of surgery, age, race, sex, body mass index, smoking status, insurance status, median income of the patient’s home zip code, American Society of Anesthesiologists (ASA) physical status classification, level of surgery, and history of spinal decompression.

Outcomes were defined as rates of 12-month revision surgery, complications, 30-day readmissions, and postoperative patient-reported outcomes (PROs). Revisions were defined as surgery performed at our institution within 12 months of the index surgery at the same spinal level excluding irrigation and debridement of soft tissues. Complications were a composite of medical (myocardial infarction and thromboembolic events), surgical (postoperative infections, hematoma, and seroma), and mechanical complications (pseudarthrosis and hardware failure). The PROs analyzed were 5-dimension European Quality of Life (EQ-5D)/Patient-Reported Outcomes Measurement Information System-Global Health (PROMIS-GH) scores, which are validated instruments for measuring several aspects of patient-reported health status, including pain, disability, functional status, and mental health.^26^

### Cost Savings

Cost savings for the 12-month periods of 2011 and 2015 were estimated and validated in a joint collaboration between the departments of supply chain management and finance at our institution. Before vendor selection, case volumes and implant needs were forecasted for the first 12 months of the contract period. Three months into the contract, these forecasts were validated and adjusted based on the first 3 months of unit volume data. Cost savings were estimated by multiplying these unit volumes by the difference between the prior prices and the newly negotiated prices. Total cost savings, savings on commodity implants, and savings on specialty and/or MIS devices were estimated and reported separately. Savings were reported only as percentages because of contractual protections between our institution and its vendors.

### Statistical Analysis

Propensity scores were estimated using separate generalized boosted models (GBMs) for each of the patient cohorts (multivendor, dual vendor, and single vendor) using the twang package in R.^27^ The GBM estimation is a machine learning technique that uses multiple regression trees in an iterative process to capture complex associations between the dependent variable and covariates without overfitting the data. The GBM automatically handles missing data, allows nonlinear associations between continuous variables and the response, and assesses for interactions. For more information on using GBMs in propensity score estimation, see McCaffrey et al.^27^ The propensity scores for each period were computed as the estimated probabilities for each associated GBM. Variables included in the propensity models included those variables previously described plus preoperative EQ-5D/PROMIS-GH utilities.

Preoperative EQ-5D/PROMIS-GH utilities were defined as the scores collected most recently before surgery but no more than 6 months before surgery. Before October 2015, our patient-reported data collection platform collected only EQ-5D scores. After October 2015, our platform switched away from EQ-5D and began collecting only PROMIS-GH data. To compare quality-of-life outcomes across the 3 periods, which span the October 2015 transition date, PROMIS-GH items were mapped to EQ-5D utilities using the linear equating methods outlined by Thompson et al.^28^ As a result, PROs are reported as a single EQ-5D/PROMIS-GH utility. This mapping method has been externally validated in patients with spinal disorders.^28^

To assess covariate balance, means (SDs) were computed for continuous variables and proportions for categorical variables. Balance between the stratified cohorts was assessed using standardized bias (absolute standardized mean difference). Covariates with a standardized bias of greater than 0.2 were considered to be imbalanced.^27^

After covariate balance was achieved, rates of revisions, complications, and readmissions were estimated by creating a logistic regression model in which the outcome was the dependent variable and patient cohort was the independent variable. This model was fit using the survey package in R to incorporate propensity score weights.^29^

To establish equivalence among these outcomes, 3 pairwise comparisons were performed for each pairing of cohorts (multivendor vs dual vendor, multivendor vs single vendor, and dual vendor vs single vendor). The null hypothesis was defined as an absolute difference of 5% or greater. The alternative hypothesis was defined as an absolute difference of less than 5%. Because of low patient numbers, the method of Gart was used to calculate a 2-tailed *P* value.^30,31^ The Fisher exact test was performed to determine whether the outcomes were significantly different from one another. Estimated outcomes and effective sample sizes, which incorporated propensity score weights, were used in these computations. Effective sample size is defined as the square of the sum of the propensity score weights divided by the sum of squared propensity score weights for each group.

For EQ-5D/PROMIS-GH utilities, we examined the change in score from before surgery to 6 months after surgery (±3 months). Means (SDs) were computed using the survey package in R to incorporate propensity score weights. To test whether the change in score was equivalent for each pairing of cohorts, the 2 one-sided tests procedure was performed using the TOSTER package in R.^32^ For each test, the upper and lower bounds were set to ±0.5 effect size units using the Cohen *d*. We also tested whether each pairwise difference in means was significantly different using a 2-sample *t* test.

All computations were done in R, version 3.6.1 (R Project for Statistical Computing), and *P* < .05 was considered to be statistically significant. The Holm method was used to correct for multiple testing of equivalence tests.^33^

## Results

### Clinical Outcomes

A total of 1373 patients (mean [SD] age, 59.2 [12.6] years; 763 [55.6%] female; 1161 [84.6%] white) were included in the study, with 272 patients in the multivendor period, 587 patients in the dual-vendor period, and 514 patients in the single-vendor period (Table 1). Before propensity score weighting, significant imbalances were observed for age, missing data for smoking status, ASA class 3, missing data for ASA, and missing data for EQ-5D/PROMIS-GH utilities. The multivendor data were characterized by younger, healthier patients (fewer rated ASA class 3) and a greater amount of missing data. Balance across all measured covariates was achieved after propensity score weighting was applied.

**Table 1.** Patient Characteristics Before and After Propensity Score Weighting^a^

Metric	Full Sample	Propensity Score Weighting					
		Before			After		
		Multivendor^b^	Dual Vendor^b^	Single Vendor^b^	Multivendor^b^	Dual Vendor^b^	Single Vendor^b^
No. or effective sample size, weighted	1373	272	587	514	177.2	546.6	418.2
Age, mean (SD), y	59.2 (12.6)	55.7 (13.5)^c^	59.4 (12.5)	60.8 (11.9)	58.6 (12.5)	59.5 (12.2)	59.6 (12.3)
Female	0.56 (0.50)	0.60 (0.49)	0.52 (0.50)	0.57 (0.49)	0.56 (0.50)	0.54 (0.50)	0.58 (0.49)
Race							
White	0.85 (0.36)	0.86 (0.35)	0.87 (0.34)	0.82 (0.38)	0.87 (0.34)	0.87 (0.34)	0.84 (0.37)
Black	0.09 (0.29)	0.10 (0.29)	0.08 (0.27)	0.10 (0.30)	0.08 (0.27)	0.08 (0.26)	0.09 (0.29)
Other	0.01 (0.12)	0.007 (0.085)	0.01 (0.12)	0.02 (0.13)	0.005 (0.068)	0.01 (0.11)	0.02 (0.13)
Missing	0.05 (0.21)	0.04 (0.19)	0.04 (0.19)	0.06 (0.24)	0.05 (0.21)	0.04 (0.20)	0.05 (0.23)
Median income, in thousands^d^							
Mean (SD), $	54.4 (16.5)	55.2 (17.8)	54.1 (16.3)	54.2 (15.9)	55.2 (16.2)	54.4 (16.1)	54.0 (15.7)
Missing	0.01 (0.10)	0.007 (0.085)	0.02 (0.13)	0.006 (0.076)	0.01 (0.11)	0.01 (0.11)	0.008 (0.088)
Insurance							
Medicaid	0.07 (0.25)	0.05 (0.22)	0.07 (0.25)	0.08 (0.27)	0.04 (0.21)	0.07 (0.26)	0.07 (0.26)
Medicare	0.48 (0.50)	0.44 (0.50)	0.50 (0.50)	0.49 (0.50)	0.47 (0.50)	0.49 (0.50)	0.47 (0.50)
Private	0.40 (0.49)	0.45 (0.50)	0.38 (0.48)	0.41 (0.49)	0.45 (0.50)	0.39 (0.49)	0.43 (0.49)
Missing	0.04 (0.20)	0.06 (0.23)	0.06 (0.23)	0.02 (0.14)	0.04 (0.19)	0.05 (0.21)	0.03 (0.18)
Smoking status							
Current	0.12 (0.32)	0.15 (0.36)	0.10 (0.30)	0.11 (0.32)	0.12 (0.32)	0.10 (0.30)	0.11 (0.31)
Former	0.45 (0.50)	0.43 (0.50)	0.46 (0.50)	0.44 (0.50)	0.43 (0.50)	0.46 (0.50)	0.44 (0.50)
Never	0.43 (0.50)	0.39 (0.49)	0.44 (0.50)	0.44 (0.50)	0.43 (0.50)	0.44 (0.50)	0.44 (0.50)
Missing	0.01 (0.10)	0.03 (0.18)^c^	0.003 (0.058)	0.004 (0.062)	0.02 (0.13)	0.006 (0.078)	0.003 (0.055)
BMI							
Mean (SD)	30.7 (6.1)	30.3 (6.4)	30.4 (5.7)	31.4 (6.4)	30.8 (6.0)	30.6 (5.9)	31.0 (6.1)
Missing	0.01 (0.09)	0.02 (0.15)	0.005 (0.071)	0.006 (0.076)	0.010 (0.099)	0.009 (0.095)	0.010 (0.099)
Prior decompression	0.24 (0.43)	0.26 (0.44)	0.26 (0.44)	0.21 (0.41)	0.27 (0.45)	0.24 (0.43)	0.22 (0.42)
Spine level							
L1-L2	0.004 (0.07)	0.004 (0.061)	0.003 (0.058)	0.006 (0.076)	0.002 (0.041)	0.003 (0.058)	0.006 (0.077)
L2-L3	0.01 (0.11)	0 (0)	0.02 (0.12)	0.01 (0.12)	0 (0)	0.01 (0.12)	0.01 (0.10)
L3-L4	0.10 (0.30)	0.08 (0.28)	0.09 (0.29)	0.11 (0.31)	0.09 (0.29)	0.09 (0.29)	0.10 (0.29)
L4-L5	0.64 (0.48)	0.62 (0.49)	0.64 (0.48)	0.65 (0.48)	0.66 (0.47)	0.65 (0.48)	0.65 (0.48)
L4-S1^e^	0.001 (0.03)	0 (0)	0 (0)	0.002 (0.044)	0 (0)	0 (0)	0.003 (0.058)
L5-S1	0.25 (0.43)	0.29 (0.45)	0.25 (0.43)	0.23 (0.42)	0.25 (0.43)	0.24 (0.43)	0.24 (0.43)
Missing	0.001 (0.03)	0.004 (0.061)	0 (0)	0 (0)	0.001 (0.034)	0 (0)	0 (0)
ASA score							
1	0.03 (0.16)	0.02 (0.13)	0.03 (0.17)	0.03 (0.16)	0.02 (0.14)	0.03 (0.16)	0.02 (0.15)
2	0.34 (0.47)	0.25 (0.43)	0.39 (0.49)	0.33 (0.47)	0.31 (0.46)	0.36 (0.48)	0.34 (0.47)
3	0.46 (0.50)	0.26 (0.44)^c^	0.48 (0.50)	0.54 (0.50)	0.46 (0.50)	0.46 (0.50)	0.49 (0.50)
4	0.02 (0.12)	0.004 (0.061)	0.009 (0.092)	0.03 (0.16)	0.006 (0.080)	0.01 (0.11)	0.02 (0.13)
Missing	0.16 (0.37)	0.47 (0.50)^c^	0.10 (0.29)	0.08 (0.27)^c^	0.20 (0.40)	0.14 (0.35)	0.13 (0.33)
EQ-5D/PROMIS-GH utility							
Mean	0.53 (0.21)	0.49 (0.22)	0.53 (0.21)	0.53 (0.20)	0.53 (0.21)	0.53 (0.21)	0.53 (0.20)
Missing	0.42 (0.49)	0.69 (0.46)^c^	0.41 (0.49)	0.29 (0.45)^c^	0.47 (0.50)	0.44 (0.50)	0.42 (0.49)

Abbreviations: ASA, American Society of Anesthesiologists physical status classification; BMI, body mass index (calculated as weight in kilograms divided by height in meters squared); EQ-5D/PROMIS-GH, 5-dimension European Quality of Life/Patient-Reported Outcomes Measurement Information System-Global Health.

^a^ Data are presented as mean (SD) percentage of patients unless otherwise indicated.

^b^ Patients were stratified by number of vendors of spinal implants available to our surgeons at the time of the patient’s surgery. January 1, 2009, to December 31, 2010, was defined as the multivendor period (10 vendors); January 1, 2011, to December 31, 2014, was defined as the dual-vendor period; and January 1, 2015, to July 31, 2017, was defined as the single-vendor period.

^c^ Standardized bias was greater than 0.2.

^d^ Median income of patient’s home zip code per US census data.

^e^ One patient had a congenital absence of the L5 vertebra.

Table 2 gives the revision, complication, and readmission rates estimated under propensity score weighting and the *P* values for each pairwise equivalence and difference test. Estimated revision rates using propensity score weighting were 3.2% (95% CI, 1.5%-6.7%) for the multivendor period, 4.5% (95% CI, 3.1%-6.5%) for the dual-vendor period, and 3.0% (95% CI, 1.7%-5.0%) for the single-vendor period. Adjusted complication rates were 5.3% (95% CI, 2.7%-10.1%) for the multivendor period, 7.2% (95% CI, 5.4%-9.6%) for the dual-vendor period, and 6.4% (95% CI, 4.6%-8.8%) for the single-vendor period. Adjusted readmission rates were 14.2% (95% CI, 9.7%-20.2%) for the multivendor period, 12.6% (95% CI, 10.1%-15.5%) for the dual-vendor period, and 9.7% (95% CI, 7.4%-12.7%) for the single-vendor period. Each pairwise equivalence test showed that revision and complication rates were statistically equivalent across the periods, with each difference in rates being less than 5% (Table 2). Furthermore, the Fisher exact test indicated a lack of significant difference in revision and complication rates (Table 2). Propensity-weighted estimates of readmissions revealed a decreasing trend during the 3 periods. None of the pairwise equivalence tests were statistically significant; thus, the null hypothesis that the difference in readmission rates was greater than 5% was not rejected. However, the Fisher exact test for each pairwise difference test indicated a lack of significant difference in readmission rates (Table 2).

**Table 2.** Revision, Complication, and Readmission Rates Before and After Propensity Score Weighting

Metric	Propensity Score Weighting^a^						*P* Value					
	Rates Before, %			Rates After, % (95% CI)			Equivalence Test^b^			Difference Test^c^		
	Multivendor (n = 272)	Dual Vendor (n = 587)	Single Vendor (n = 514)	Multivendor (n = 177.2)^d^	Dual Vendor (n = 546.6)^d^	Single Vendor (n = 418.2)^d^	Multi vs Dual Vendor	Multi vs Single Vendor	Dual vs Single Vendor	Multi vs Dual Vendor	Multi vs Single Vendor	Dual vs Single Vendor
Revisions	2.9	4.8	2.9	3.2 (1.5-6.7)	4.5 (3.1-6.5)	3.0 (1.7-5.0)	.02	.04	<.001	.43	.87	.21
Complications	4.4	7.5	7.4	5.3 (2.7-10.1)	7.2 (5.4-9.6)	6.4 (4.6-8.8)	.02	.04	.003	.40	.62	.58
Readmissions	13.6	13.1	10.9	14.2 (9.7-20.2)	12.6 (10.1-15.5)	9.7 (7.4-12.7)	.06	.89	.57	.58	.11	.14

^a^ Patients were stratified by number of vendors of spinal implants available to our surgeons at the time of the patient’s surgery. January 1, 2009, to December 31, 2010, was defined as the multivendor period (10 vendors); January 1, 2011, to December 31, 2014, was defined as the dual-vendor period; and January 1, 2015, to July 31, 2017, was defined as the single-vendor period.

^b^ Equivalence test *P* values were calculated under the null hypothesis that the pairwise difference between rates was greater than 5%. Equivalence test *P* values were corrected for multiple testing using the Holm method.

^c^ Difference test *P* values were calculated under the null hypothesis that rates were equal.

^d^ The n value is the effective sample size of the population after applying propensity score weights.

The mean changes in EQ-5D/PROMIS-GH utilities after propensity score weighting were 0.20 (95% CI, 0.13-0.27) for the multivendor period, 0.21 (95% CI, 0.17-0.24) for the dual-vendor period, and 0.19 (95% CI, 0.16-0.22) for the single-vendor period. Each pairwise equivalence test showed that mean changes in EQ-5D/PROMIS-GH scores were statistically equivalent (*P* = .002 for multivendor vs dual vendor, *P* = .003 for multivendor vs single vendor, and *P* < .001 for dual vendor vs single vendor) across the periods. In addition, none of the pairwise difference tests were statistically significant (*P* = .92 for multivendor vs dual-vendor, *P* = .70 for multivendor vs single vendor, and *P* = .42 for dual vendor vs single vendor). When comparing patients who did and did not have data for change in EQ-5D/PROMIS-GH, patients who were missing data vs those not missing data were significantly more likely to be in the multivendor period (multivendor, 25.3%; dual vendor, 43.0%; and single vendor, 31.7%; *P* < .001), were more likely to be missing the ASA score (24.6% vs 2.3%, *P* < .001), were younger (mean [SD] age, 58.2 [13.0] years vs 60.9 [11.7] years; *P* < .001), and lived in zip codes with lower median income (mean [SD], $53 700 [$17 000] vs $55 500 [$15 400]; *P* = .045). All other measured covariates were not significantly different.

### Cost Savings

In 2011, adoption of a dual-vendor strategy for the purchase of commodity spinal implants was associated with a 24% reduction in the cost of commodity devices. The capitated pricing scheme for the purchase of specialty and/or MIS devices was associated with a 35% reduction in the annual expense of these devices. Together, these cost containment efforts were associated with a 25% decrease in the overall expense of spinal implants.

 In 2015, transition to a sole vendor of commodity implants was associated with a 21% reduction in the cost of commodity products. Advancement of the capitated pricing scheme was associated with a 2% decrease in the cost of specialty and/or MIS devices. In total, these initiatives were associated with a 15% decrease in the total annual expenditure on spinal implants, improving on the 25% cost savings created previously in 2011.

## Discussion

The importance of cost consciousness in health care is widely acknowledged by physicians.^34,35^ How to reduce the cost of care and with whom the responsibility lies, however, are questions not easily answered. The significant financial burden of orthopedic implants is well understood by surgeons, and prior research^35^ suggests that many surgeons agree that limiting vendors to reduce hospital expenditures is appropriate for hospitals and physicians. In practice, however, physicians have strong vendor loyalties and tend to resist policies that limit or dictate implant choice.^24,35^ One explanation for such ambivalence is that relatively few surgeons are affected by administrative measures that restrict implant choice.^35^ Preserving physician autonomy in implant selection is a key feature of many cost-saving initiatives that target medical devices.^12,13,14,15,16,17,18,19,20,21,22,23,24^ The limitation of such choice-preserving measures is, however, that manufacturers often require volume-based guarantees to justify meaningful price reductions.^19,24,25^ As a result, hospitals may be forced to balance the competing priorities of reducing cost and protecting physician choice. As described in this study, our approach to resolving this conflict was to consolidate vendors of products that were determined to be fungible (commodities) and to protect physician autonomy in using novel or proprietary devices (specialty and/or MIS devices). Because more than 70% to 80% of our expenditures on implants go toward the purchase of commodity devices, we believed this strategy could yield significant financial gains with minimal disruptions to the practice of our surgeons.

Physician adherence to programs that target commodity products is highly dependent on whether the participating surgeons agree that the devices are fungible. As a result, an important aspect of our initiative was to encourage physician participation in the process, which has been noted in prior literature^19^ to be a key component of other successful cost-saving programs. Physician participation also helped ease surgeons’ distrust in the process and spare hospital-physician relationships, which are positively correlated with hospitals’ ability to contain costs.^36^

Physician preferences for items such as spinal implants are based on a variety of considerations, including relationships with vendor sales representatives, sales and training support, personal experience with a device or brand, and cost and other financial considerations.^37^ Nevertheless, technological factors, such as patient outcomes, implant longevity, ease of use, and implant design, are most important in shaping physician preferences for orthopedic implants.^37^ Therefore, any initiative that curtails access to physician preference items has the possibility of affecting patient outcomes and should be evaluated not only on the economic merits of the program but also on its potential clinical impact.

In this study, use of a sole source of commodity implants was not associated with a significant difference in the rates of 12-month revision surgery, perioperative complications, or 30-day readmissions for patients undergoing single-level lumbar interbody fusion. In addition, single-vendor procurement was not associated with differences in patient-reported health status as measured by EQ-5D/PROMIS-GH utilities. Furthermore, rates of revisions, complications, and PROs were statistically equivalent across the multivendor, dual-vendor, and single-vendor periods.

The economic benefit of consolidating our vendor base from 10 suppliers to 2 was a 24% savings in the annual expense of commodity spinal implants. Four years later, advancing to a single-vendor program was associated with an additional 21% in savings. In comparison, the economic benefit of capitated pricing negotiations was 35% in 2011 and 2% in 2015. The 2011 and 2015 processes were nearly identical; the primary difference was the number of vendors contracted to provide commodity implants. However, in 2015, the marginal benefit of vendor rationalization was greater than that of capitated pricing. Many explanations likely account for these observations, not least of which is the pricing power expected from developers of novel products. However, we believe that they are partly explained by the considerable value that volume-based incentives have for device manufacturers.

In 2017, two years after implementing our cost-saving initiative, the terms of our single-vendor contract were renegotiated. Our institution was able to obtain an additional 12% reduction in the price of commodity implants, defying conventional wisdom that reliance on a sole manufacturer undermines the economic benefits of price competition among multiple vendors.^25^ The economic results reported in this study must be interpreted with caution, however, because our experience may not be generalizable, especially to lower-volume institutions.

### Limitations

This study has several limitations. Our assessment of surgical quality is based on a retrospective analysis, which is inherently subject to bias. In addition, our analysis only evaluated rates of revisions, complications, readmissions, and PROs for patients undergoing single-level lumbar interbody fusion. Although we believe that this population and these metrics provide a good barometer for the overall quality of care at our institution, we cannot be sure that use of single-vendor procurement did not compromise other unmeasured areas of quality. Moreover, our study only evaluated short-term outcomes that occurred within 12 months of surgery; long-term outcomes were not evaluated.^19^

In addition, the PROs data that we analyzed consisted of 2 different scales collected at different times. Although we used a published mapping algorithm to convert PROMIS-GH scores to EQ-5D utilities, our results may have been different if we had collected the same scale across the entire study period. We also may have been underpowered to detect differences in binary outcomes, particularly given the smaller size of the multivendor cohort. All pairwise comparisons were within 5% of each other, however, which was our threshold for determining equivalence. We recommend future studies with larger sample sizes to confirm our results.

## Conclusions

This study showed that single-vendor procurement of spinal implants was associated with significant cost savings for our institution. In addition, transition to single-vendor procurement was not associated with a decline in our quality of care, as measured by rates of revision surgery, complications, and 30-day readmissions, as well as patient-reported health status. Our surgical quality as defined by these metrics was statistically unchanged whether a multivendor, dual-vendor, or single-vendor procurement model was used.
